# Interaction with macrophages attenuates equine fibroblast‐like synoviocyte ADAMTS5 (aggrecanase‐2) gene expression following inflammatory stimulation

**DOI:** 10.1002/jor.23891

**Published:** 2018-04-24

**Authors:** Rhiannon E. Morgan, Peter D. Clegg, John A. Hunt, John F. Innes, Simon R. Tew

**Affiliations:** ^1^ The Royal Veterinary College Equine Referral Hospital Hawkshead Lane Hatfield AL9 7TA; ^2^ Department of Musculoskeletal Biology Institute of Ageing and Chronic Disease The University of Liverpool William Henry Duncan Building West Derby Street Liverpool Merseyside L7 8TX; ^3^ Institute of Veterinary Sciences The University of Liverpool, Leahurst Chester High Road Neston Cheshire CH64 7TE

**Keywords:** synovium, macrophage, aggrecanase, ADAMTS5

## Abstract

The joint synovium consists of a heterogeneous cell population, chiefly comprised of macrophages, and fibroblast‐like synoviocytes (FLS). An inter‐species co‐culture model was developed to examine interactions between these cells. Equine FLS and the canine macrophage line DH82 were differentially labeled using fluorescent markers and results from direct co‐culture compared with those from both indirect co‐culture, and conditioned media experiments. The transcript expression of IL‐1β, IL‐6, ADAMTS4, and ADAMTS5 in each cell type were determined using species‐specific qPCR assays. Lipopolysaccharide stimulation of EFLS rapidly increased IL‐1β, IL‐6, ADAMTS4, and ADAMTS5 mRNAs. The induction of ADAMTS5 was significantly reduced when equine FLS were cultured with DH82 cells directly or indirectly. Exposure of equine FLS to denatured conditioned media also significantly reduced ADAMTS5 induction. DH82 cells increased interleukin‐1β expression substantially following LPS stimulation. However, knockdown of interleukin‐1β in DH82 cells, or inhibition of NF‐κB in equine FLS prior to co‐culture did not change the inhibitory effect on equine FLS ADAMTS5 gene expression. This work indicates that macrophages can influence FLS gene expression through a soluble mediator, and modulate the expression of an enzyme critical in osteoarthritis pathology during inflammatory stimulation. © 2018 The Authors. *Journal of Orthopaedic Research*® Published by WileyPeriodicals, Inc. on behalf of the Orthopaedic Research Society. J Orthop Res 36:2178–2185, 2018.

The synovial membrane is a specialized tissue that lines non‐articulating surfaces of diarthrodial joints. It contains a population of fibroblast‐like synoviocytes in addition to monocytic cell populations, predominantly macrophages. Synovitis is a key mediator of osteoarthritis,^1^ and is involved in the perpetuation of cartilage degradation.^2^ Synovial inflammation is characterized by mononuclear cell infiltration^3^ and increases in catabolic cytokines, especially IL‐1β and TNF‐α,^4^ which are predominantly produced by the synovial macrophages. These cytokines regulate fibroblast‐like synoviocyte gene expression, including those encoding cartilage matrix degrading proteinases.^5^ Examples of these include the ADAMTS (a disintegrin and metalloproteinase with thromboSpondin motif) family of enzymes, particularly ADAMTS4 and ADAMTS5, which can cleave the major cartilage proteoglycan aggrecan with the latter being heavily implicated in the development of cartilage degeneration during osteoarthritis^6^, ^7^ Although constitutively expressed in human chondrocytes and osteoarthritic synovial cells, there is evidence that murine and bovine chondrocyte ADAMTS5 mRNA levels respond to treatment with IL‐1β.^8^ This may be mediated through NF‐κB signaling, with a recent study identifying the transcription factor RelA/p65 as a potent transcriptional activator of ADAMTS5 in chondrocytes during osteoarthritis.^9^ In addition to transcriptional regulation, ADAMTS5 can be controlled at the post‐translational level^8^ and recent work has identified LRP‐1‐mediated endocytic clearance of active ADAMTS5 as a key regulatory process.^10^

Like humans, the horse is susceptible to chronic joint diseases such as osteoarthritis with associated synovial inflammation^11^ and equine synovium expresses both ADAMTS4 and 5.^12^ Interestingly, normal equine fibroblast‐like synoviocytes (EFLS) expressed significantly lower levels of ADAMTS5 mRNA when co‐cultured with injured cartilage in comparison to co‐culture with normal cartilage.^13^ These factors suggest that EFLS may have a protective influence during joint inflammation, although how fibroblasts and macrophages in the synovium interact is poorly understood.

In this study, an in vitro co‐culture system was created to allow interactions between EFLS and macrophages to be identified. EFLS proliferative and gene regulation responses to inflammatory stimulation and to contact with macrophages was analyzed.

## METHODS

### Isolation of Equine Fibroblast‐Like Synoviocytes (EFLS)

Forelimbs from three horses were collected from a local abattoir. The metacarpophalangeal joint was cleaned and skinned before opening. The joint surface was scored macroscopically to ensure absence of orthopaedic disease.^3^ Synovium was dissected, finely diced, and cells isolated using 0.25% (w/v) trypsin/0.1% (w/v) EDTA digestion followed by incubation with 2 mg/ml collagenase type 2 (Worthington Biochemicals; Berkshire, UK) Cells were seeded at 6 × 10^4^ cells/cm^2^ and expanded for up to three passages using split ratios of 1:3. Unless stated otherwise, all cells were cultured in Dulbecco's Modified Eagle Medium (Life Technologies; UK) containing 10% foetal bovine serum (FBS), 0.2% amphotericin B (2 µg/ml), and 1% penicillin (100 U/ml) streptomycin (100 µg/ml) at 37°C in 5% CO_2_.

### Western Blot Analysis

EFLS (*n* = 3) cells were cultured with and without 10µg/ml LPS (E. Coli 026:B6, Sigma–Aldrich, Poole, UK) for 6 or 16 h. Culture media was collected, the cells washed using PBS, and lysed in 1× SDS sample‐loading buffer (62.5 mM Tris–HCl, pH 6.8 at 25°C, 2% w/v SDS, 10% glycerol, 50 mM DDT, 0.01% w/v bromophenol blue, or phenol red). Cell lysate and culture media protein concentrations were measured using the Pierce™ 660 Protein Assay (Life Technologies; Carlsbad, CA). Protein (40 µg) was extracted from each culture media sample using StrataClean™ Resin according to manufacturer protocol and eluted by boiling in 15 µl 1× SDS sample buffer. Cell lysate and culture media protein samples were run on SDS‐PAGE gels and blotted onto nitrocellulose membranes, which were then probed using a sheep anti‐mouse ADAMTS5 antibody (a kind gift from Amanda Fosang, Melbourne Australia)^14^ at a dilution of 1:500 followed by an anti‐goat/sheep IgG‐peroxidase secondary antibody at a dilution of 1:1000 (A9452; Sigma–Aldrich). Antibody localization was visualized using Western Lightening‐Plus chemiluminescence reagent (Perkin Elmer, Beaconsfield, UK), and imaged on a UVP ChemiDoc‐it imaging system. Intensity of bands was quantified using imageJ software.

### Multi Species Fibroblast‐Like Synoviocyte/Macrophage Co‐Culture Model

The canine macrophage‐derived cell line DH82 was obtained from American Tissue Culture Collection (ATCC, Manassas, VA) and cultured under the same conditions as the EFLS. Fluorescently labeled DH82 cells were produced by infecting them with a lentivirus expressing green fluorescent protein (GFP) (Sigma) and subsequent selection with 2 µg/ml puromycin (Life Technologies). EFLS were labeled with 8 µM Cell Proliferation Dye eFluor**^®^** 670 (eBioscience Inc., San Diego, CA) for 10 min using the manufacturers protocol. Cells were mixed and seeded in 6‐well dishes at 3.3 × 10^4^cells/cm^2^ at the following EFLS:DH82 ratios: (i) 1:0; (ii) 0:1; (iii) 1:2; (iv) 2:1; (v) 1:1. Co‐cultures were analyzed using an Accuri™ C6 flow cytometer (BD Biosciences, Oxford, UK).

### Design, Validation, and Testing of Species‐Specific qPCR Assays

Equine and canine mRNA sequences for the following genes; GAPDH, IL‐1β, IL‐6, ADAMTS4, and ADAMTS5, were obtained from the NCBI GenBank database (Table 1). Equine and canine mRNA sequences were directly compared using NCBI BLAST to determine dissimilar regions, to which primers were designed. All primers were designed using Primer Express 2.0 software (Applied Biosystems, UK) and synthesized by Eurogentec (Seraing, Belgium). PCR efficiencies of primer pairs were determined using serial 2‐fold dilutions of species‐appropriate cDNA.

**Table 1:** Species‐Specific Primer Sequences and Gene Accession Numbers for Sequences Used in Their Design

Gene	Gene Accession No.	Primer Sequence
*Equus caballus* GAPDH	NM_001163856.1	F: TGACCCCCTAACATATTGAGAGTCT
		R: GCCCCTCCCCTTCTTCCTG
*Equus caballus* IL‐1β	NM_001082526.1	F: GAGCCCAATCTTCAACATCTATGG
		R: ATACCAAGTCCTTTTACCAAGCCTG
*Equus caballus* IL‐6	NM_001082496.1	F: CCTGGTGATGGCTACTGCTTTC
		R: GGATGTACTTAATGTGCTGTTTGGTT
*Equus caballus* ADAMTS4	NM_001111299.1	F: CAGCCTGGCTCCTTCAAAAA
		R: ATGTGGTCACTATTCCTGCGG
*Equus caballus* ADAMTS5	XM_003364218.2	F: ACCGATCCTGCAGTGTCACA
		R: AAATCTTTTCGCCATGAGCAG
*Canis lupus* GAPDH	NM_001003142.1	F: AACTGCTTGGCTCCTCTAGCC
		R: CCACGATGCCGAAGTGGT
*Canis lupus* IL‐1β	NM_001037971.1	F: CTATCATCTGCAAAACAGATGCG
		R: GCATGGCTGCATCACTCATAAA
*Canis lupus* IL‐6	NM_001003301.1	F: CCTGGTGATGGCTACTGCTTTC
		R: TGGCATCATCCTTGGAATCTC

F, forward sequence; R, reverse sequence; all sequences are shown 5′–3′.

### EFLS and DH82 Co‐Cultured in Direct Contact

EFLS (*n* = 3) and DH82 cells were seeded independently or mixed 1:1 at 1.5 × 10^5^/cm^2^. Cells were allowed to attach overnight and then exposed to 10 µg/ml LPS, and harvested 1, 3, 6, 12, and 24 h later. Where necessary, DH82 cells at 150,000 cells/well of a 6‐well plate, were transfected with 25 pmol canine anti‐IL‐1β siRNA or silencer Cy3 labeled negative control siRNA (csiRNA) (Life Technologies) using the lipofectamine 2000 (Life Technologies) prior to co‐culture, or EFLS were pre‐treated for 1‐h with either 0.8 µM Bay‐11‐7082 (B5556; Sigma–Aldrich, Missouri) or 200 μM Pyrrolidinedithiocarbamic acid (PDTC; Enzo Life Sciences, New York,NY).

### EFLS and DH82 Co‐Cultured Without Direct Contact

DH82 cells were stimulated with 10 μg/ml LPS for 24 h and the conditioned media collected and added to EFLS cells seeded at 5 × 10^4^cell/well in 24 well plates. To examine the effect of protein denaturation, conditioned media was heated to 100°C for 10 min prior to use. Compartmentalized culture was performed by seeding 2 × 10^4^ DH82 cells into 0.45 µm cell culture inserts (Merck Millipore, Darmstadt, Germany) for 24 h, before transferring the insert to a well containing a monolayer of EFLS before stimulation with 10 µg/ml LPS for 6 and 12 h.

### Reverse Transcription and Quantitative RT‐PCR

Total RNA (1 µg), purified using the Guanidinium‐thiocyanate‐phenol‐chloroform technique,^15^ was reverse transcribed using M‐MLV reverse transcriptase (Promega, WI) using random primers. qRT‐PCR was performed with 160 ng cDNA using SYBR Green master mix (GoTaq qPCR Master Mix, Promega) and 300PromeganM individual primer concentrations on an ABI 7300 instrument. The relative quantification of each gene normalized to GAPDH was calculated using the 2^−ΔCt^ method.^16^

### Statistical Analysis

Two tailed *t*‐tests were used to compare fold changes in EFLS mRNA expression when cultured independently in standard culture media, compared to those cultured with DH82, in conditioned media, in denatured conditioned media, and with DH82 cells in well‐inserts, and also to compare the gene expression of DH82 cells cultured alone and in co‐culture with EFLS. Student *t*‐tests with bonferroni post hoc corrections were used to compare DH82 gene expression when treated with control siRNA compared to anti‐IL‐1β siRNA, and EFLS ADAMTS5 gene expression when cultured with DH82 cells either treated with control siRNA or anti‐IL‐1β siRNA, or when pre‐treated with NF‐κB inhibitors.

## RESULTS

### LPS Stimulated EFLS Secreted Increased Levels of ADAMTS5 Protein

**Figure 1:**
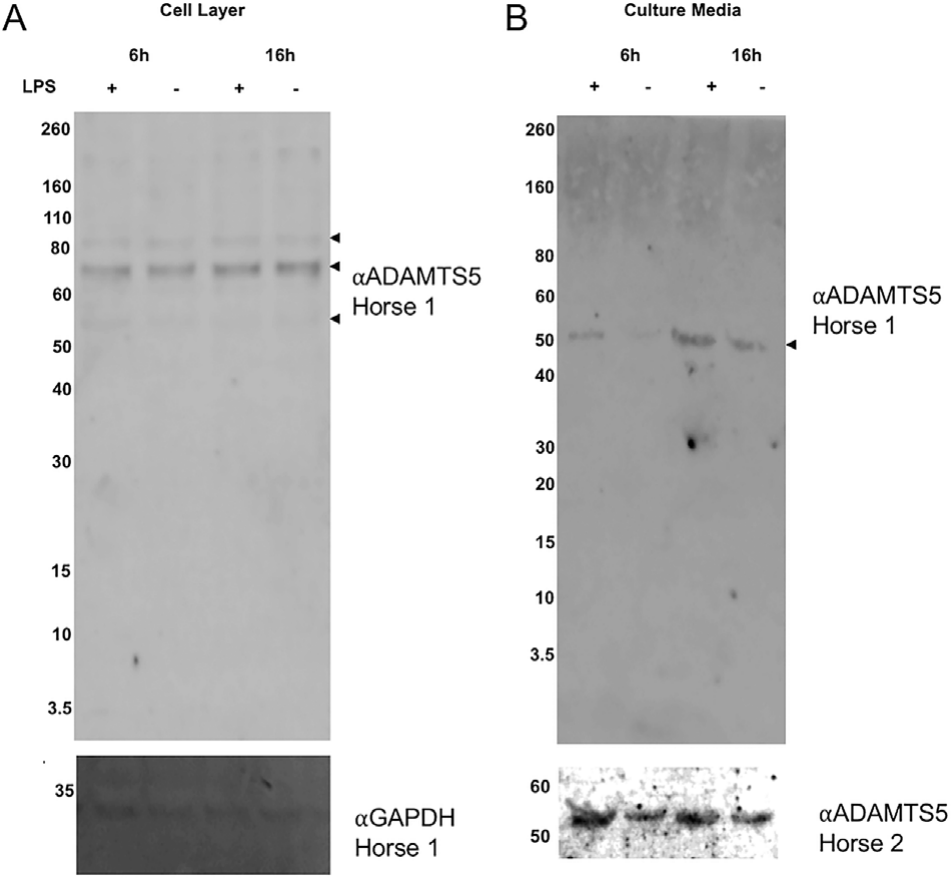
Expression of ADAMTS5 in EFLS cell layer and culture media. EFLS were cultured in the presence or absence of 10 μg/ml LPS. Western blotting was performed on (A) cell layer harvested from one horse and (B) culture media harvested at 6 and 16 h post‐LPS exposure from two different horses, using an antibody which recognizes ADAMTS5. GAPDH was used as a loading control for the cell layers (A). Forms of ADAMTS5 with molecular weights of 75, 100, and 55–60 kDa were detected within the cell layers, while a 55 kDa form was detected in EFLS culture media (indicated by arrowheads in A and B).

We were interested in the levels of the aggrecanase ADAMTS5 in the cell layer and culture media of EFLS exposed to inflammatory simulation. Western blot analysis of cell lysates identified bands at approximately 90 and 70 kDa (Fig. 1A). The predicted molecular weight of the pro form of equine ADAMTS5 is 101 kDa. There was no LPS mediated induction of ADAMTS5 protein in the cell layer fraction after 6 h of treatment, although levels were slightly increased in both control, and LPS treated cells after 16 h (1.3× and 1.4× increase respectively, compared to 6‐h control conditions). We detected an approximately 50 kDa band, in western blot analysis of EFLS media (Fig. 1B), which potentially corresponds to ADAMTS5 which has undergone auto‐catalytic removal of the C‐terminal domains.^14^ After 6 h, levels of the 50 kDa ADAMTS5 were 2.9 times higher than controls. Examination of media after 16 h of culture again revealed higher levels of 50 kDa ADAMTS5 in LPS treated cells (2× higher than 16‐h control conditions). Upon repeating, using cells from a second horse, we again observed more intense 50 kDa ADAMTS5 immunopositive bands in LPS treated cells at both time points (Fig. 1B).

### Macrophages Stimulate EFLS Mitosis

**Figure 2:**
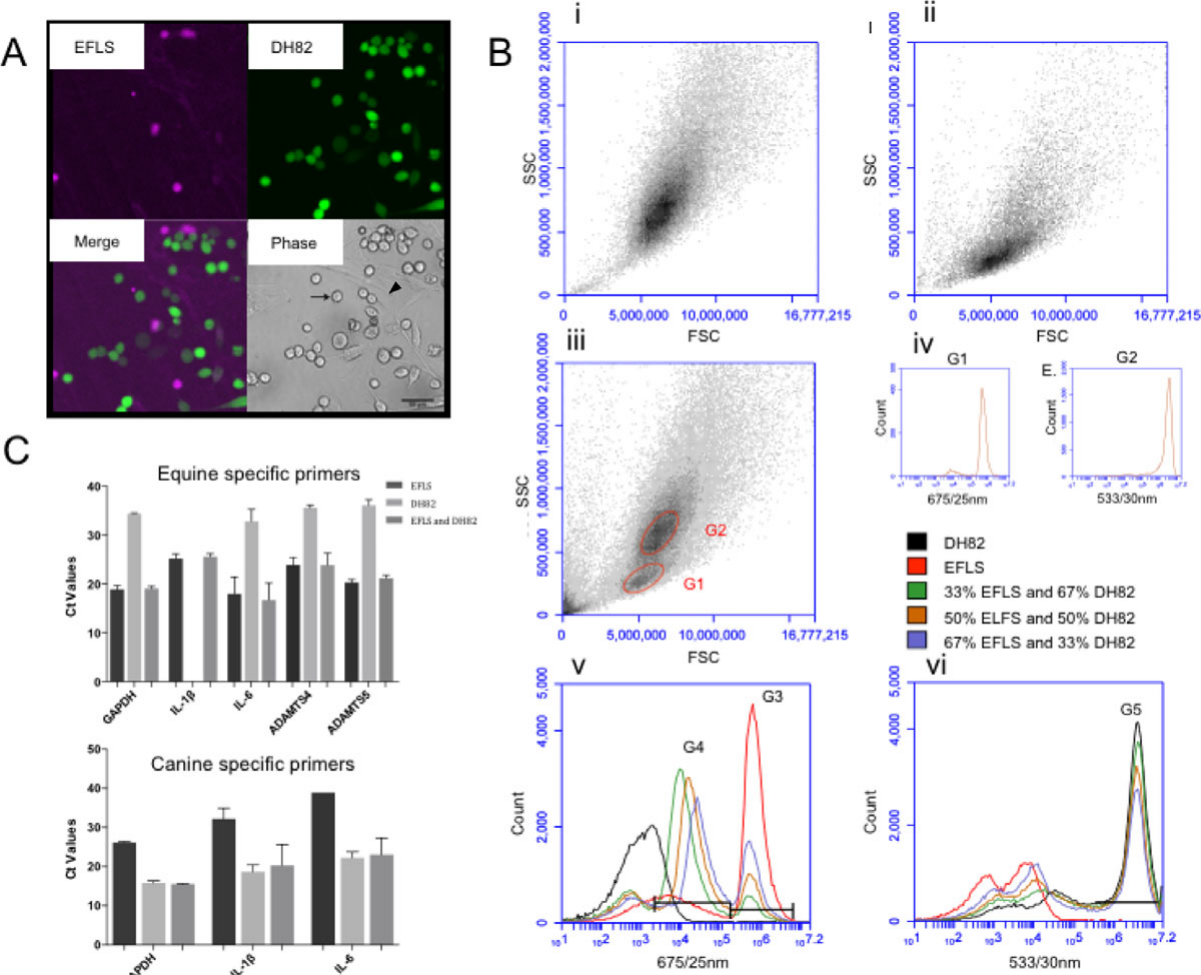
Characterization of EFLS/DH82 co‐culture system. (A) Confocal microscopy of GFP‐expressing DH82 and Efluor‐labeled EFLS cells in 1:1 co‐culture. Efluor‐labeled EFLS (purple) were excited using a far red (633 nm) laser line and detected with a 660/20 nm band pass filter. GFP‐expressing DH82 cells (green) were excited using a green (488/532 nm) laser line and detected with a FITC band pass filter. An overlay image of both channels is shown as well as a phase contrast image of DH82 (arrow) and EFLS (arrowhead) cells in co‐culture. (B) Flow cytometry analysis of GFP‐expressing DH82 and efluor‐labeled EFLS cells in co‐culture. Density plot diagrams of DH82 (i) and EFLS (ii) cultured individually and in co‐culture (iii); EFLS (G1) and DH82 (G2) populations were gated. (iv) Fluorescence intensity emitted from cells within the gated areas (G1 and G2). (v and vi). Fluorescence intensity emitted by labeled DH82 and EFLS after 24 h in co‐culture at different proportions, detected by two different interference filters 675/25 nm (v, red) and 533/30 nm (vi, green), respectively. Gates corresponding to EFLS primary (G3) and secondary (G4) generations, and DH82 cells (G5) were applied to enable cell numbers within each co‐culture to be determined. (C) Average qRT‐PCR cycle threshold (Ct) values for each equine and canine sequence primer pair, when used to measure expression in DH82, EFLS, or co‐cultures (*n* = 3). Ct values were higher, reflecting low expression of genes, when primers of one species were applied to cDNA from the second species. Error bars represent SEM.

Given that EFLS could upregulate ADAMTS5 in response to an inflammatory stimulation, we wanted to determine how EFLS ADAMTS5 response was affected by interactions with the inflammatory cells that they would encounter in vivo. To do this, we generated a multi‐species co‐culture model which could allow investigation of cell interactions whilst permitting specific measurement of EFLS response. We fluorescently labeled EFLS with the Cell Proliferation Dye eFluor**®** 670 and generated non‐clonal, canine DH82 macrophage cell cultures that expressed GFP, which enabled discrimination of the cells when cultured together (Fig. 2A). Using microscopy and flow cytometric analysis, we observed a slightly higher rate of EFLS proliferation compared to DH82 cells, when co‐cultured over 24 h. Side scatter (SSC) versus forward scatter (FSC) density plots identified two groups of cells with differing morphological characteristics within the mixed culture. After analysis of individual cultures, EFLS were identified to be smaller and less granular than DH82 cells (Fig. 2B(i) and (ii)).

When the two populations within the mixed culture were gated on the SSC vs FSC density plot (G1 = EFLS, G2 = DH82) (Fig. 2B(iii)), the majority of cells within the G1 gated area were consistent with efluor‐labeled EFLS (Fig. 2B(iv)), while those within the G2 gated area were consistent with GFP‐expressing DH82 cells (Fig. 2B(iv)), demonstrating that the two populations could be independently assessed in the mixed cultures.

Proliferation rates of each population in co‐culture were analyzed. A slightly higher rate of EFLS proliferation vs. DH82 proliferation, during 24 h co‐culture was observed, and EFLS mitosis was stimulated by co‐culture with DH82 cells (Fig. 2B(v) and (vi)). When EFLS were cultured independently, 67% of the cells were labeled with the efluor dye (Fig. 2B(v)). As cells undergo mitosis, the dye is distributed equally between the two daughter cells, and this can be measured by the fluorescence intensity halving. Within mixed co‐cultures, fluorescence intensity of the eFluor dye identified two sub‐populations, emitting fluorescence intensity at 10^5.3^–10^6.7^ or 10^3.3^–10^5.3^, consistent with primary and secondary generations of EFLS, (Fig. 2B(v)). This secondary EFLS population was not identified over the same time scale when EFLS were cultured alone, indicating that the presence of DH82 cells stimulates EFLS mitosis.

The number of labeled DH82 and EFLS cells within each co‐culture was analyzed to calculate the proportion of each population after 24 h (Fig. 2B(v) and (vi)). The initial seeding ratio of 50% EFLS and 50% DH82 produced a culture of 60% EFLS and 40% DH82 after 24 h. An infiltration of cells into the synovial membrane, resulting in 25–50% of the synovial cell population consisting of mononuclear cells, has been classified as a moderate (grade 3 out of 4) synovitis (McIlwraith and Frisbie^3^). This proportion of cells was therefore deemed appropriate to model a moderate synovitis after 24 h in culture.

### Production of Species‐Specific qRT‐PCR Assay

To allow us to specifically measure cell‐specific gene expression in co‐cultures, we designed species‐specific qRT‐PCR assays. Primers deemed to be species‐specific when they measured robust expression, reflected by low Ct values when used to analyze expression cultures of the same species (alone or in co‐culture), but exhibited high or undetectable Ct values when used to analyze expression in cultures just containing the other species’ cells (Fig. 2C).

We also ensured that dissociation curves of PCR products displayed a single peak when analyzing cultures that contained cells of the target species as the primers, but were either not evident or negligible when primers were used on cultures solely containing cells of the other species. Discriminatory assays were not always possible to generate however, meaning that specific analysis of ADAMTS4 and ADAMTS5 in DH82 cells was not carried out as repeated iterations of primer design failed to identify a specific assay.

### Macrophages Attenuate EFLS ADAMTS5 Gene Expression

**Figure 3:**
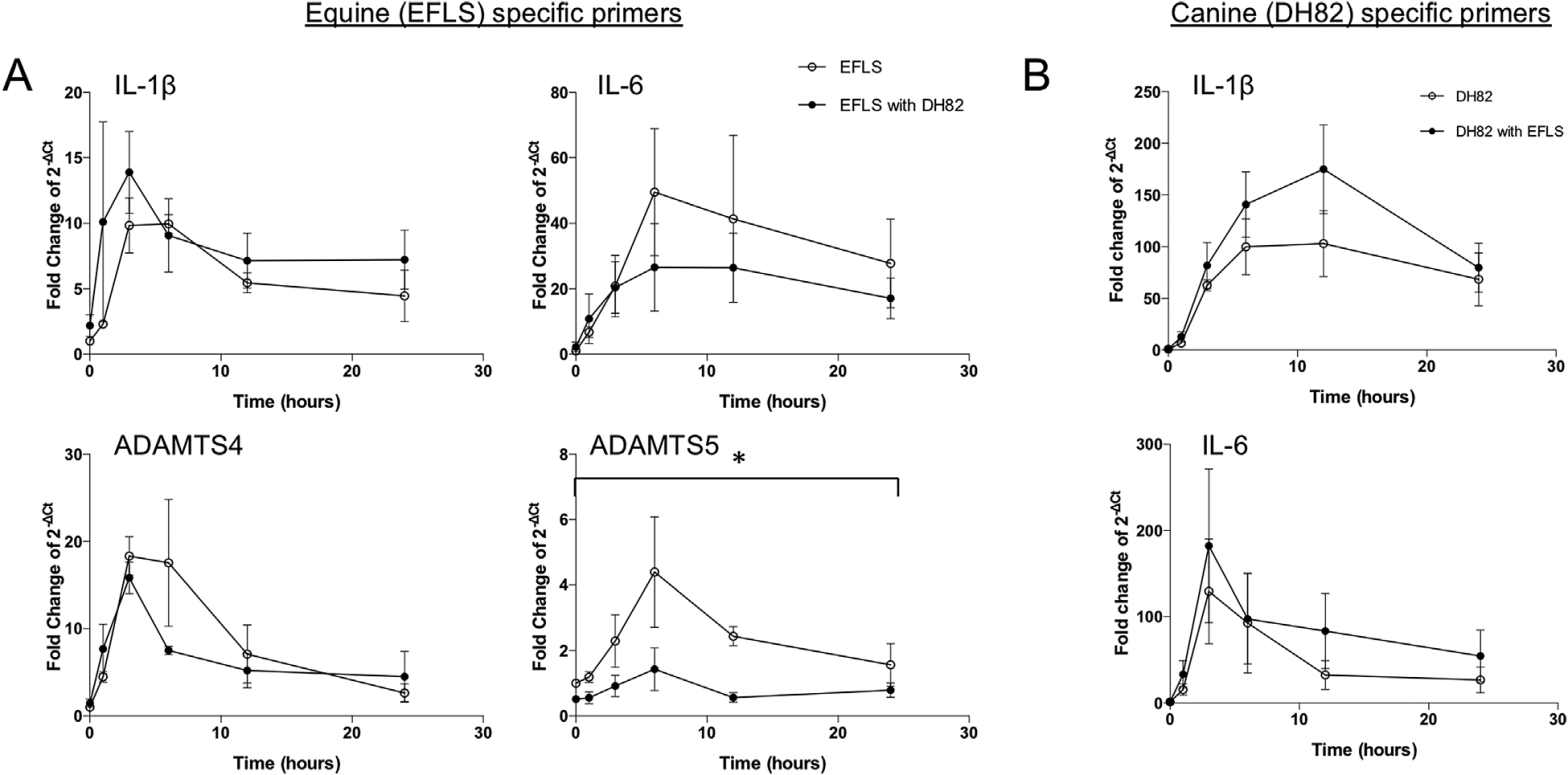
Fold changes of EFLS gene expression when cultured independently or in co‐culture with DH82 macrophages. (A) Gene expression of IL‐1β, IL‐6, ADAMTS4, and ADAMTS5 measured by qRT‐PCR in EFLS when cultured independently or in co‐culture with DH82. (B) Gene expression of IL‐1β and IL‐6 gene measured by qRT‐PCR in DH82 cells when cultured independently or in co‐culture with EFLS (*n* = 3). Data are displayed as fold changes (*n* = 3) compared to the gene expression of independently cultured EFLS at 0 h with no LPS exposure. Gene expression was measured at 1, 3, 6, 12, and 24 h post‐LPS exposure. Error bars represent SEM. Increased expression of ADAMTS5 mRNA over the 24‐h period was significantly (*p < 0.05) lower when EFLS were cultured with DH82.

EFLS cultured independently demonstrated rapid increases in IL‐1β, IL‐6, ADAMTS4, and ADAMTS5 mRNAs in response to 10 µg/ml LPS (Fig. 3A). Peak expression of each mRNA occurred 3–6 h post‐LPS exposure. Maximum fold changes in mRNA levels of EFLS cultured independently and with DH82 cells respectively, were 10× (at 6 h) and 14× (at 3 h) for IL‐1β mRNA, 50× (at 6 h), and 27× (at 6 h) for IL‐6 mRNA, 18× (at 3 h) and 16× (at 3 h) for ADAMTS4 mRNA, and 4× (at 6 h) and 1.4× (at 6 h) for ADAMTS5 mRNA. Interestingly, the increased expression of ADAMTS5 mRNA over this time period was significantly (*p* < 0.05) lower in EFLS that were co‐cultured with DH82 cells. Although ADAMTS4 and IL‐6 mRNA levels tended to be lower when EFLS were in co‐culture, these differences were not statistically significant.

### DH82 Macrophage Gene Expression When Co‐Cultured With EFLS

DH82 cells cultured independently or in co‐culture with EFLS, demonstrated a reproducible, rapid increase in the expression of the cytokines IL‐1β and IL‐6 mRNA following exposure to LPS (Fig. 3B). Peak fold change in IL‐6 gene expression occurred earlier than IL‐1β (3 and 12 h, respectively), and the mean expression of both cytokines was higher when co‐cultured with EFLS, although this was not statistically significant.

### Macrophage Attenuation of EFLS ADAMTS5 is Not Driven by IL‐1β Expression

**Figure 4:**
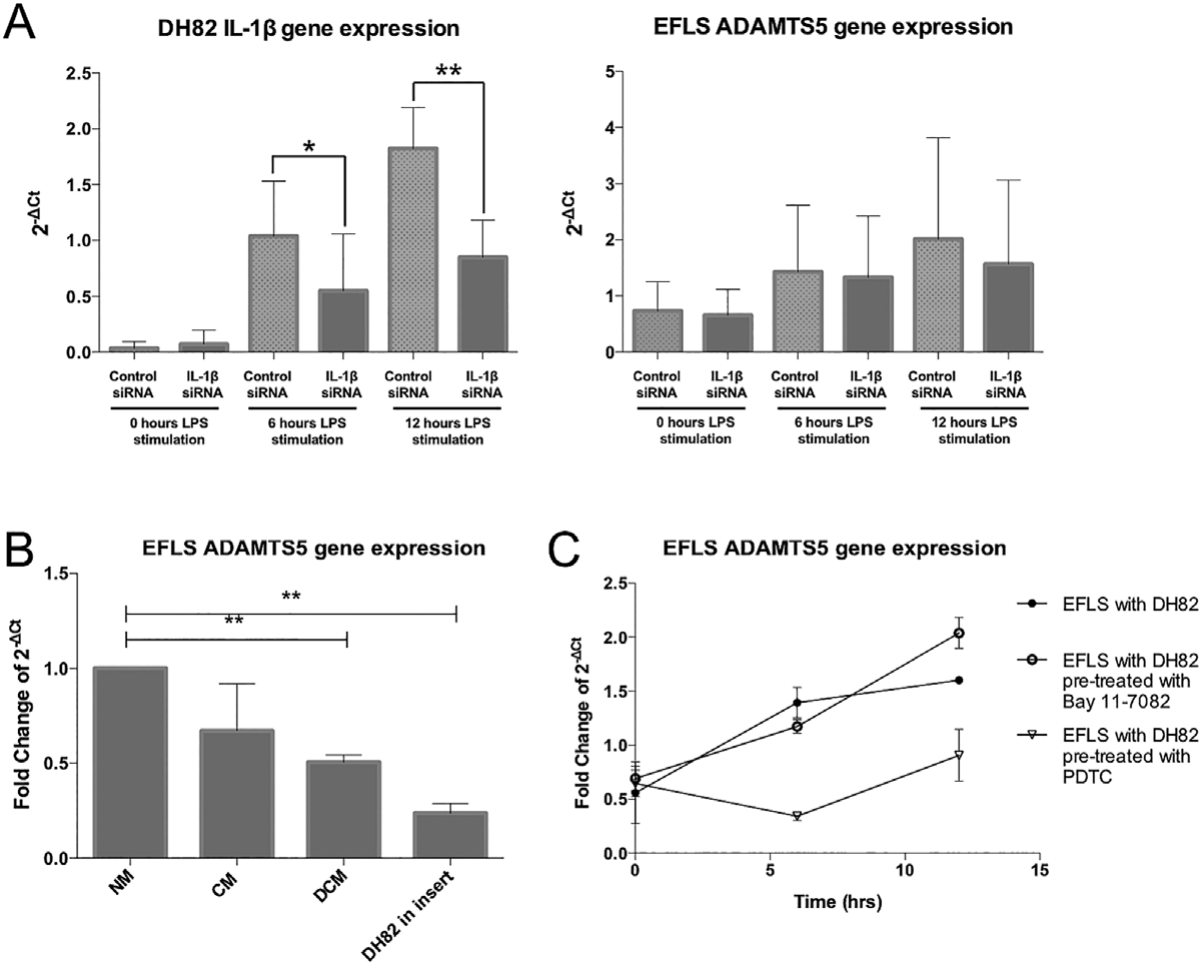
Mechanistic investigation of EFLS ADAMTS5 mRNA attenuation by DH82 cells. (A) DH82 were transfected with either csiRNA or with canine anti‐IL‐1β siRNA and co‐cultured with EFLS (*n* = 3) in the presence of 10 μg/ml LPS. Histograms display DH82 IL‐1β gene expression and EFLS ADAMST5 gene expression. (B) EFLS ADAMTS5 gene expression when cultured in DH82 conditioned media, or with DH82 cells within well‐inserts. Fold changes in EFLS ADAMTS5 gene expression are compared to EFLS cells cultured in LPS‐containing normal media (NM). EFLS expression when cultured with DH82 conditioned media (CM) and DH82 denatured conditioned media (DCM) (**p < 0.01), and with DH82 cultured in well inserts (**p < 0.01) is displayed. All conditions (*n* = 3) were harvested 12 h after 10 ug/ml LPS exposure. (C) Fold changes in EFLS ADAMTS5 gene expression after pre‐treatment with NF‐κB inhibitors. EFLS (*n* = 3) in direct co‐culture with DH82 cells were pre‐treated with the NF‐κB inhibitors; Bay 11‐7082 and PDTC before exposure to 10 μg/ml LPS. Cells were harvested at 0, 6, and 12 h post‐LPS exposure. Data is presented as fold changes in EFLS ADAMTS5 gene expression compared to the gene expression of EFLS cells in co‐culture at 0 h. All data in this figure is presented as mean ± SEM, *p < 0.05, **p < 0.01

When IL‐1β binds to the IL‐1 membrane bound receptor, it activates several pathways including the NF‐κB pathway. RelA/p65, an NF‐κB family member, is a strong transcriptional activator of ADAMTS5 in chondrocytes.^17^ It was hypothesized that increased DH82 IL‐1β gene expression may influence EFLS ADAMTS5 gene expression. Statistically significant down‐regulation of macrophage IL‐1β gene expression was achieved at 6h (*p* < 0.05) and 12 h (*p* < 0.01) post‐LPS exposure using siRNA designed to target canine IL‐1β compared to a non‐specific control siRNA (Fig. 4A). Co‐culture of IL‐1β knockdown DH82 cells EFLS did not change the inhibitory effect of the macrophages on the induction of ADAMTS5 mRNA following LPS stimulation, indicating that macrophage‐derived IL‐1β expression is not responsible for controlling this inhibition (Fig. 4A).

### A Soluble Mediator Produced by Macrophages is Responsible for the Attenuation of ELFS ADAMTS5 Gene Expression

EFLS cultured in LPS‐containing DH82 conditioned media (CM) expressed lower levels of ADAMTS5 mRNA (Fig. 4B) compared with those cultured in LPS‐containing unconditioned media (NM). This suggests that the attenuation of ADAMTS5 in the co‐culture system is mediated by a soluble factor. Interestingly, EFLS expression of ADAMTS5 mRNA was also lower when the cells were cultured in denatured conditioned media (DCM) (*p* < 0.01), indicating that this soluble factor is thermally stable (Fig. 4B). Consistent with these findings, indirect co‐culture culture of EFLS with DH82 using well inserts, also led to repressed ADAMTS5 mRNAs (*p* < 0.01) (Fig. 4B).

### Co‐Culture Does Not Influence EFLS Through NF‐κB Signaling

To investigate the influence of the NF‐κB signaling pathway on EFLS ADAMTS5 gene expression, EFLS in co‐culture with DH82 cells were pre‐treated with NF‐κB inhibitors before being exposed to LPS (Fig. 4C). Neither inhibitor prevented the suppression of ADAMTS5 induction in co‐cultures. The presence of the inhibitor PDTC led to an overall suppression of EFLS ADAMTS5 gene expression levels, while the inhibitor Bay 11‐7082 led to similar levels of ADAMTS5 mRNA compared to non‐treated EFLS in co‐culture. None of these effects were statistically significant.

## DISCUSSION

Synovial fibroblasts are a potential source of the catabolic factors that contribute to joint tissue breakdown during diseases such as osteoarthritis. Previous studies have identified that inflammatory mediators can control the expression of a range of proteinases by these cells. This study aimed to determine whether there was cross talk between synovial fibroblasts and macrophages, using a novel, multi‐species culture model. The role of macrophages within the inflammatory response to joint injury has been investigated using murine macrophage depleted experimental OA models. A reduction in osteophyte formation, fibrosis, synovial activation, synovial lining growth factor production, MMP‐induced neoepitope formation, and synovial MMP3 and −9 mRNA expression, were observed in macrophage‐depleted subjects, suggesting that synovial macrophages are moderators of OA joint pathology.^18^ However, macrophage‐depletion in OA models, has also been linked to increased acute joint inflammation involving significantly higher synovitis scores, increased cellular density, and a reduction in bone mineral density.^19^, ^20^ Macrophages are obviously pivotal in the synovial response to joint injury, and provide essential moderation of joint inflammation. Understanding how macrophages interact with other cells within the synovium should be valuable, given the emerging role of synovitis in osteoarthritis pathology.

We initially observed that ADAMTS5 protein secretion was increased by EFLS following stimulation with LPS. Various catalytically active isoforms of the ADAMTS5 protein have been observed by other groups. We observed a variety of immunopositive bands present in the cell layers of EFLS but only one major band in the media fraction, which migrated at 50 kDa. This could be analogous to a similarly sized form missing the C‐terminal cysteine rich and spacer domains but still retaining the pro‐domain which was observed by Kosasih et al.^14^ This would suggest that the most abundant isoform of ADAMTS5 secreted into the media by EFLS is not active. We should not currently rule out the possibility of other isoforms being present in the EFLS culture media however, as we used a precipitation method to concentrate the secreted protein which may have led to non‐uniform isoform enrichment.

Having observed that LPS stimulation of EFLS led to an increase in their secretion of ADAMTS5 protein, we decided to further examine the regulation of ADAMTS5 in our co‐culture system. Although ADAMTS5 has multiple tiers of control, many of which occur post‐translationally, we found that mRNA was induced over a similar time scale to its protein secretion. This supported the use of mRNA expression to determine ADAMTS5 response in a cell‐specific manner in our co‐cultures, taking advantage of species‐specific PCR primers for transcript quantification. We used the canine DH82 cell line in this study. These cells were originally derived from a histiocytic sarcoma and share many properties with canine macrophages,^21^ although they do not appear to be strongly polarized toward either M1 or M2 macrophage subtypes.^22^

One major finding of this study is that co‐culture with macrophages promotes proliferation of EFLS. When EFLS were co‐cultured with DH82 cells, loss of fluorescence intensity of the eFluor dye occurred in proportion to the number of DH82 cells present, indicating that EFLS mitosis was stimulated by the co‐culture of DH82 cells. Macrophage migration inhibitory factor (MIF) has been shown to significantly stimulate the proliferation of rheumatoid arthritis FLS, with indirect IL‐1β and TNF‐α involvement.^23^ IL‐1 is also known to stimulate FLS proliferation.^24^ As macrophages are major producers of both IL‐1 and MIF, these factors may explain the influence that DH82 cells have on EFLS proliferation and this may be relevant to the synovial hyperplasia observed during osteoarthritis. EFLS ADAMTS5 gene expression was significantly attenuated when cells were co‐cultured with DH82 macrophages (*p* < 0.05).

A further finding of the study was that co‐culture of EFLS with macrophages inhibits inflammatory‐mediated stimulation of ADAMTS5 expression in the same cells. Induction of ADAMTS5 gene expression by LPS was also suppressed when EFLS were exposed to macrophage‐conditioned medium, and even more strongly when EFLS cells were cultured with macrophages indirectly using well inserts. The suppressive effect of DH82 conditioned medium was also evident when it had been heat denatured. A soluble factor released by macrophages, which is either thermally stable or can reversibly denature, is likely to be responsible for these findings. We had wanted to determine whether IL‐1β mediated the process as its expression by macrophages was higher when they were co‐cultured with EFLS. However, siRNA‐mediated knockdown of IL‐1β in DH82 cells did not affect their ability to attenuate EFLS ADAMTS5 expression in co‐culture. This observation is consistent with a previous study which reported that EFLS ADAMTS5 gene expression did not respond to IL‐1β stimulation alone.^25^ We could also not demonstrate a specific role for NFkB signaling in the modulation of EFLS response by macrophage conditioned media. Equine synoviocyte ADAMTS5 mRNA was reduced when normal synoviocytes were cultured with injured cartilage, compared to normal cartilage,^13^ a process that was proposed to be a mechanism to protect injured cartilage. Therefore, crosstalk clearly exists between fibroblast‐like synoviocytes and other cells within joint tissues and further work is needed to determine the mechanisms that underpin this.

## AUTHORS’ CONTRIBUTIONS

REM and SRT contributed to all sections. PDC and JAH contributed to the conception and design of the study, the analysis of data, drafting, and final approval of the article. JFI contributed to the conception and design of the study, and final approval of the article. All authors have read and approved the final submitted manuscript.
